# Mohs Micrographic Surgery for Dermatofibrosarcoma Protuberans in 15 Patients: The University of Arkansas for Medical Sciences Experience

**DOI:** 10.7759/cureus.24147

**Published:** 2022-04-14

**Authors:** Blake St. Clair, Abigale Clark, Benjamin Rollins, Thomas A Jennings

**Affiliations:** 1 Department of Dermatology, University of Arkansas for Medical Sciences, Little Rock, USA; 2 Department of Dermatology, Kansas City University College of Osteopathic Medicine, Kansas City, USA; 3 Department of Pathology, University of Arkansas for Medical Sciences, Little Rock, USA

**Keywords:** dermatofibrosarcoma protuberans, dermatopathology, mohs micrographic surgery, sternum, glabella, mohs surgery, mms, dfsp

## Abstract

Background: Dermatofibrosarcoma protuberans (DFSP) is an uncommon, locally aggressive malignancy with wide local excision (WLE) or Mohs micrographic surgery (MMS) representing the treatment of choice. This article illustrates the experience of a single academic institution in treating DFSP with MMS and adds two particularly large, difficult closures of the glabella/central forehead and sternum to the body of literature.

Objective: To report the results of 15 patients with DFSP treated with MMS over a five-year period by a single Mohs surgeon at the University of Arkansas for Medical Sciences (UAMS).

Methods: A total of 15 patients between the ages of 16 and 80 years were diagnosed with DFSP and treated with MMS and were contacted in October 2021 to assess for recurrence.

Results: None of the 15 patients had a recurrence of DFSP following MMS, with a mean follow-up interval of 22.4 months and an average of 1.93 Mohs layers required for tumor clearance.

Conclusion: This experience reaffirms that MMS is an effective treatment for DFSP and adds additional examples of closure techniques of large, ovoid surgical defects on the glabella/central forehead and sternum to the literature.

## Introduction

Dermatofibrosarcoma protuberans (DFSP) is a rare, low-grade, locally aggressive malignant tumor that typically presents as an indurated plaque on the trunk or upper limbs [1]. Its prevalence is estimated at approximately 4.2 cases/million people per year in the United States [2]. It is a slow-growing, insidious tumor that frequently has a delayed diagnosis as it may mimic non-malignant processes such as a keloid, dermatofibroma, or epidermal cyst [3]. DFSP is associated in over 90% of cases with a t(17;22) translocation that produces a platelet-derived growth factor (PDGF)-beta/collagen type 1A1 fusion gene [2]. This PDGF-beta protein acts as a potent mitogen for PDGF receptor beta [4], which is a tyrosine kinase and is the basis for the rationale for using imatinib, a tyrosine kinase inhibitor, in inoperable/locally advanced or metastatic DFSP, and potentially in a neoadjuvant setting.

Wide local excision (WLE) (with at least 2-3 cm margins extending at least to fascia) and Mohs micrographic surgery (MMS) have been established as the treatment modalities of choice for DFSP [5,6]. Targeted therapy with imatinib and radiotherapy are also utilized in specific clinical scenarios. Several studies have also shown that the risk of recurrence is lower and the size of post-operative defects is smaller in lesions treated with MMS rather than WLE [5,7,8], though either technique is a reasonable approach assuming full histologic margins are assessed.

Here, we summarize the clinical course of 15 patients with DFSP treated with MMS and highlight reconstruction techniques of the forehead and sternum for two particularly large and infiltrative cases of DFSP.

## Materials and methods

This was a retrospective study of patients diagnosed with DFSP by board-certified dermatopathologists and treated with MMS by a single fellowship-trained Mohs surgeon at the University of Arkansas for Medical Sciences. The electronic medical records of 15 patients treated for DFSP with MMS between August 2016 and September 2021 were identified. This was accomplished by querying the institution’s electronic medical record pathology database for dermatopathology specimen final diagnoses mentioning “dermatofibrosarcoma protuberans” or “DFSP,” then filtering these results for dermatopathology specimens submitted by the Mohs surgeon (all DFSP tumors treated with MMS had central debulking sections submitted for permanent histologic processing).

In each case, the diagnosis of DFSP was confirmed prior to surgical removal and again confirmed on final re-excision. All but three cases were evaluated for the presence vs. absence of fibrosarcomatous change. MMS was performed using an initial margin of 1 cm from the clinically palpable tumor. If needed, additional Mohs layers were taken with an additional 1-cm margin. As noted above, the central debulking sections were submitted for permanent histological processing. Mohs layers with questionable pathology were also submitted for permanent histological processing with routine hematoxylin and eosin staining. No other staining including immunohistochemical staining was performed. The wounds were repaired through linear closure in 12 cases, a purse-string closure in one case on the abdomen, an advancement flap in one case on the glabella/central forehead, and a split-thickness skin graft in one case on the sternum. The latter two reconstructions were performed by an otolaryngologist and a plastic surgeon, respectively, at the same academic institution.

Demographics that were recorded included patient age, gender, and ethnicity. Tumor characteristics that were documented included the location of the tumor and the presence vs. absence of fibrosarcomatous change on permanent histopathologic slides. Treatment outcomes that were recorded included the number of Mohs stages required for clearance, the size of post-surgical defects, follow-up interval (time elapsed between surgery and October 2021), and whether or not tumor recurrence was noted. All patients were contacted by telephone in October 2021 to ask about tumor recurrence. Tumor recurrence was assessed by asking patients whether they had any new tumors, nodules, scarring, pain, or any other changes at their surgery sites. Moreover, 12/15 patients had been evaluated in the dermatologic surgery clinic within the year prior to October 2021 with no clinical evidence of recurrence noted by the Mohs surgeon. All patients included in this study provided verbal consent for participation.

## Results

Of the 15 cases of DFSP treated with MMS, none reported recurrence with a mean follow-up time of 22.4 months (range: 1-59 months). No patients in our cohort received imatinib or adjuvant radiotherapy. The average age of the patient population was 41.6 years (range: 16-80 years) and 10 (66.7%) patients were females and five (33.3%) were males. Additionally, it was observed that two cases occurred in Hispanic patients, nine were in Caucasian patients, and four were in Black patients.

As is typical of DFSP, the trunk was the most common location in our cohort with 10 of the 15 cases (66.6%) arising here. An additional two cases occurred on the upper limb, two on the lower limb, and one case occurred on the forehead/glabella.

The average number of Mohs stages required to clear the tumors was 1.93 (range: 1-3), with an average post-operative size (smallest x largest dimension) of 5.7 x 9.38 cm (Table 1). Of the 12 cases closed with linear closure, the average length of the closure was 12 cm. The remaining cases were closed with a split-thickness skin graft, advancement flap, and a purse-string closure, respectively.

**Table 1:** Clinical and histopathological characteristics, reconstruction, and follow-up.

Characteristic	The University of Arkansas for Medical Sciences cohort
Mean number of Mohs stages to clear (1 cm margins)	1.93
% with fibrosarcomatous change	13.3% (2/15)
Mean post-operative defect size	7.3 x 5.9 cm
Reconstruction technique	Primary closure in 12 cases (mean length: 12 cm), purse-string closure in one case, split-thickness skin graft in one case, and advancement flap in one case
Mean follow-up time	22.4 months (range: 1-59 months)
% with recurrence at the time of follow-up	0% (0/15)

**Figure 1:**
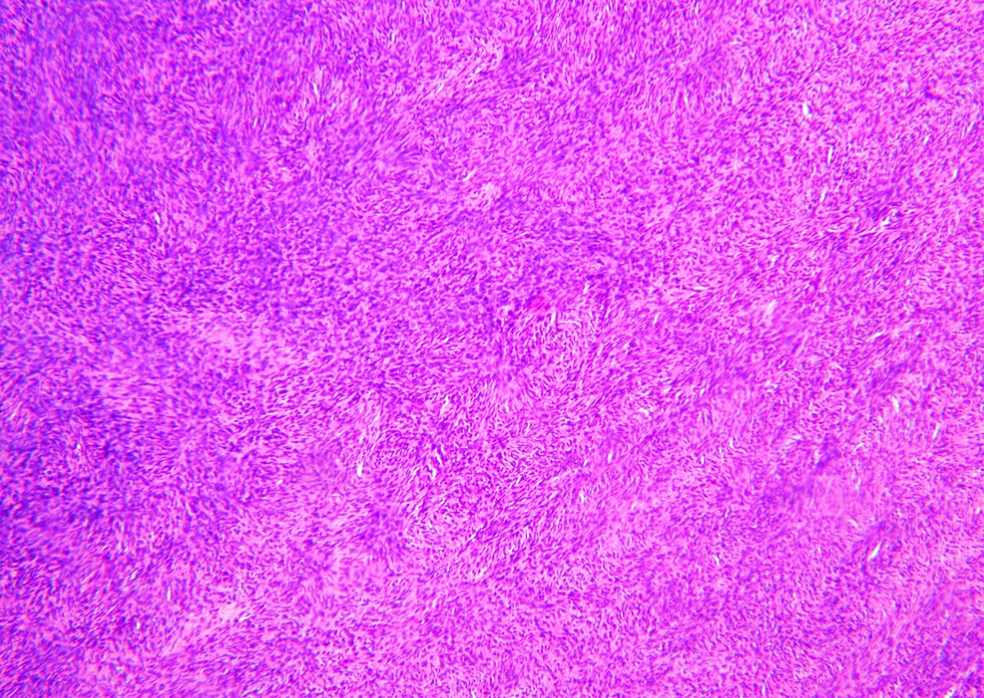
Dermatofibrosarcoma protuberans with fibrosarcomatous transformation. This low power view of a case in our series demonstrates the gradual and sometimes subtle progression from the conventional storiform pattern of dermatofibrosarcoma protuberans (top left) to the more fascicular or herringbone pattern of growth seen with fibrosarcomatous transformation (bottom right). Hematoxylin and eosin, 100x magnification.

Fibrosarcomatous change was noted on histopathologic examination in 2/15 (13.3%) patients (Figure 1), whereas 10/15 (66.7%) patients did not demonstrate fibrosarcomatous change. The pathologist had no comment on whether fibrosarcomatous change was observed in the remaining three patients. Interestingly, one patient had a non-contiguous soft tissue mass on the shoulder biopsied at the time of DFSP diagnosis that resulted in a spindle-cell lipoma.

Of the 15 cases, only one case on the glabella/forehead represented recurrent DFSP (recurred twice after two prior WLE) while the remaining 14 cases were consistent with primary DFSP.

**Figure 2:**
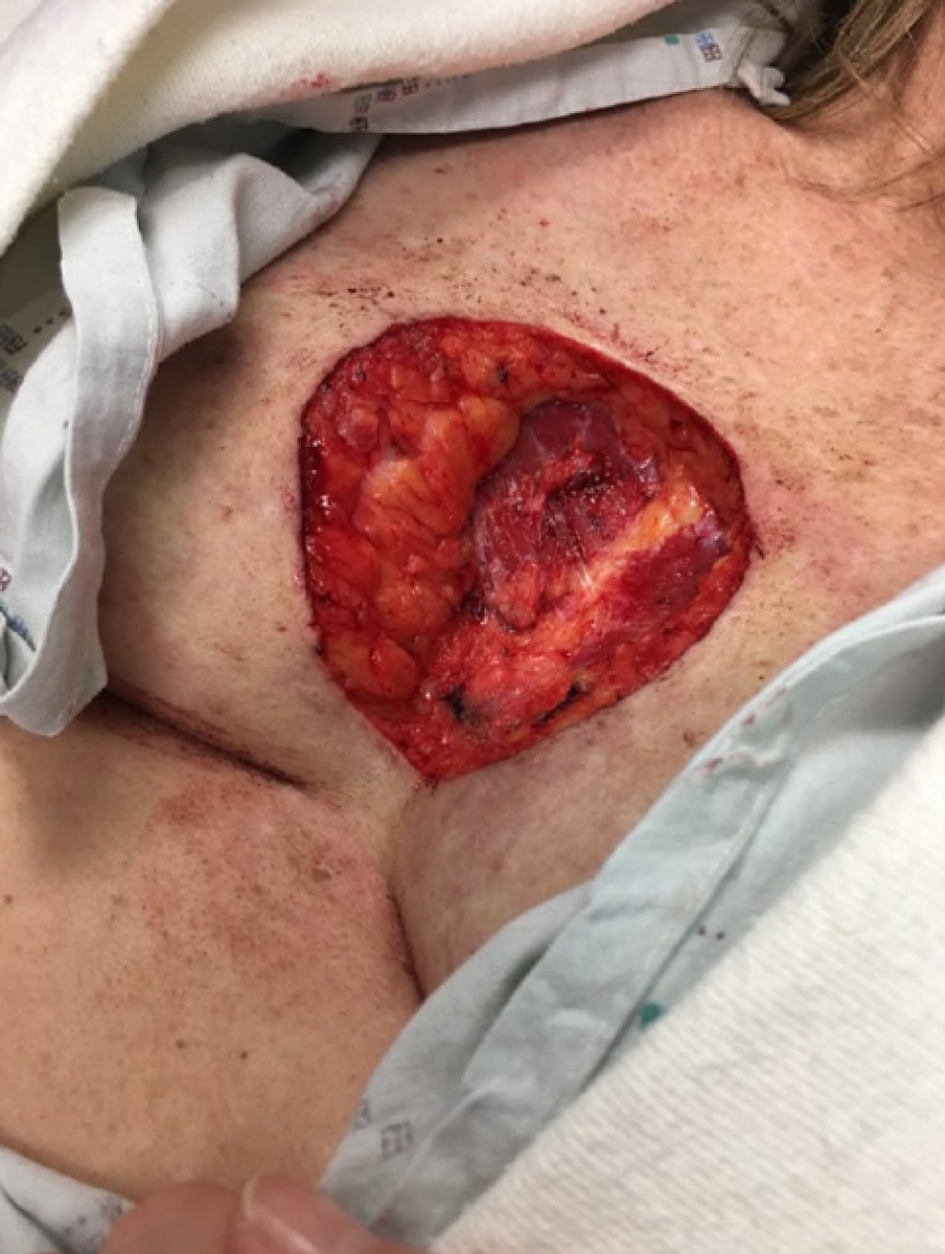
Dermatofibrosarcoma protuberans on the sternum treated with Mohs micrographic surgery. The tumor was deceptively infiltrative with a final defect size of 6.2 x 10.3 cm.

**Figure 3:**
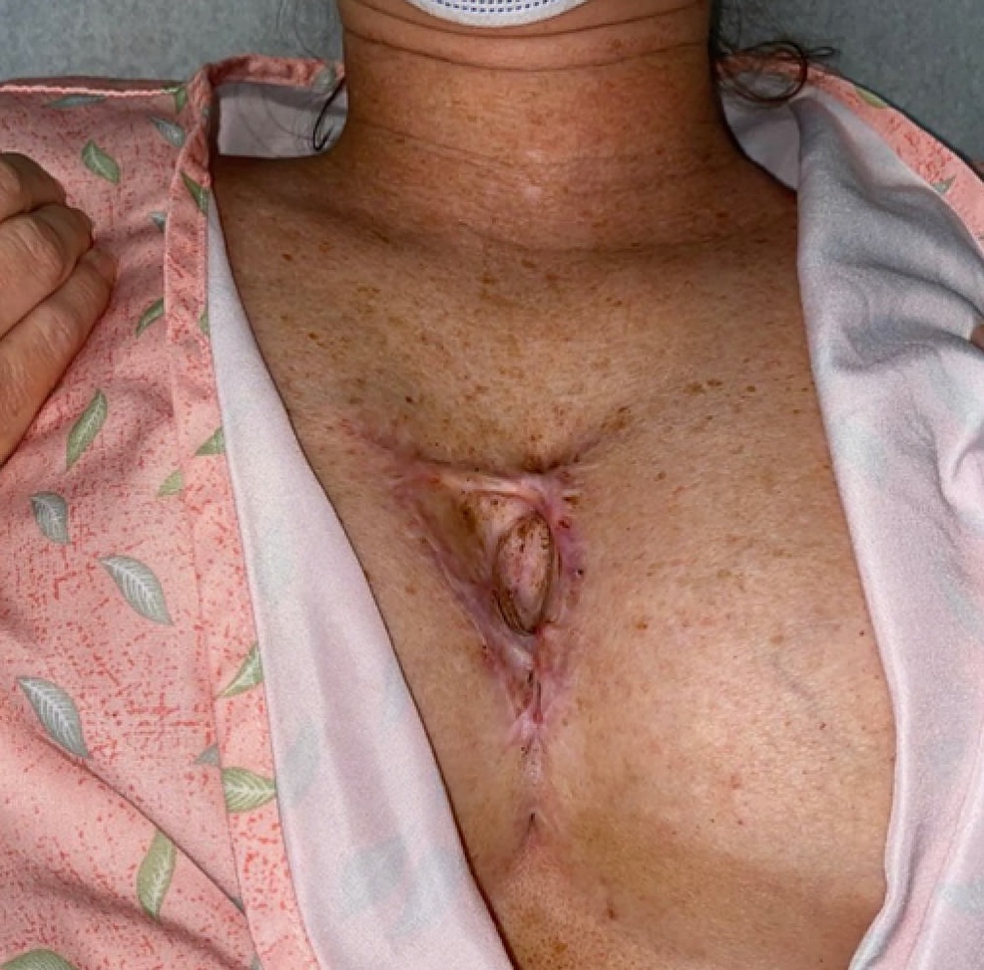
Dermatofibrosarcoma protuberans on the sternum. One-year post-operative photograph following reconstruction with a split-thickness skin graft.

In our case series, two cases, in particular, had challenging reconstructions due to the large size of the post-operative defects and the location of the malignancies. A 48-year-old Caucasian woman had a deceptively infiltrative DFSP over the sternum with a post-operative defect size of 6.2 x 10.3 cm (Figure 2). In this case, the patient was referred to plastic surgery and temporarily repaired with a split-thickness graft harvested from the thigh with plans for a more extensive reconstruction after two years of tumor-free survival. A post-operative photograph at one year is shown in Figure 3.

**Figure 4:**
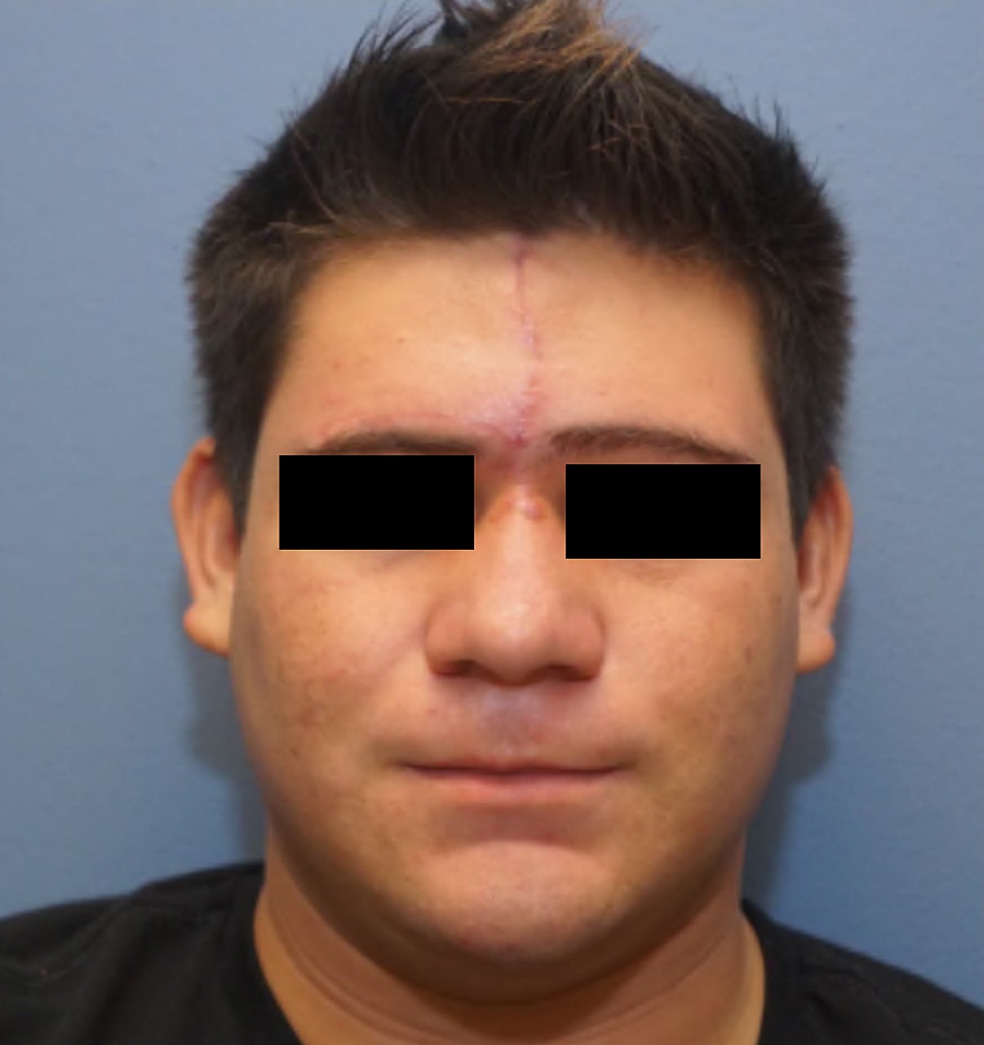
Dermatofibrosarcoma protuberans on the glabella/central forehead. One-month post-operative photograph following reconstruction with a right-sided advancement flap.

In the other case, a 16-year-old Hispanic male had a large, multiply recurrent, infiltrative DFSP of the glabella and central forehead with a final defect size of 5.2 x 5.5 cm. The patient was referred to facial plastics for reconstruction and the defect was repaired using a right-sided advancement flap. To achieve this, a Burrow’s triangle was removed in the right eyebrow. A W-plasty along the nasion region of the nose was performed, and tacking sutures were used to hold down deep soft tissue from the left side of the defect where the missing frontalis muscle has been resected. Once these steps were accomplished, the flap was advanced and closure was performed. A post-operative photograph at two months is shown in Figure 4.

## Discussion

DFSP is an uncommon, low-grade sarcoma with a high propensity for local recurrence but relatively low metastatic potential. Surgical removal with either MMS or WLE is the treatment of choice for DFSP with recent practice trending toward surgical removal with MMS, and while no randomized control trial has been performed, it has been estimated in one large review that the recurrence rate in MMS vs. WLE is 1.3% and 20.7%, respectively [9]. Case series similar to ours in which patients were treated with MMS have shown similar demographics, tumor characteristics, and low recurrence rates [1,10-12]. Beyond surgical removal, imatinib is a small-molecule tyrosine kinase inhibitor that was FDA-approved in 2006 for the treatment of metastatic, recurrent, or unresectable DFSP that functions as an effective PDGF receptor inhibitor. Combinations of imatinib followed by MMS have been employed to treat recurrent DFSP [13]. Not all DFSPs respond well to imatinib, particularly translocation negative DFSP and pigmented DFSP (Bednar tumor) [14]. Radiation therapy may be used as definitive therapy for nonsurgical candidates, as adjuvant radiotherapy in cases with positive margins or with recurrent disease, and as palliative radiotherapy for incurable disease or disease with extensive metastases [15].

While the 10-year survival for DFSP in the United States has previously been estimated at 99.1%, risk factors including increased age, male sex, Black race, DFSP location on the limbs or head, and large tumor size have all been associated with poorer outcomes [2,16]. Additionally, the presence of fibrosarcomatous change has been shown to be a clear risk factor for aggressive behavior. In a review of 1422 patients with DFSP, 225 patients with fibrosarcomatous change had a local recurrence rate of 29.8%, metastasis in 14.4% of cases, and death from disease in 14.7% in contrast to a local recurrence rate of 13.7%, metastasis in 1.1%, and death from disease in 0.8% of cases in the remaining “classic” DFSP cases [17]. Several additional studies have reinforced this higher risk fibrosarcomatous subtype and noted that they generally are larger tumors that require more Mohs stages to clear and often have extensive subcutaneous infiltration [18,19]. As noted above, two of the 15 patients in this series had fibrosarcomatous change associated with their DFSPs. Neither of these patients had a recurrence of their DFSPs to date.

The clinical differential diagnosis of DFSP can be broad with keloids, neurofibromas, leiomyomas, epidermal cysts, melanoma, morpheaform basal, dermatofibromas, and other mimickers often entering the differential diagnosis and requiring a histologic evaluation to parse the difference. Histologically, DFSP is characterized by a dense collection of bland cells with spindle-shaped nuclei arranged into irregular, interwoven fascicles in the dermis that often have a storiform appearance [20] with invasion into the fat and subsequent entrapment of adipocytes in a “honeycombing” pattern. It should be noted that other spindle cell entities can mimic DFSP histologically and DFSP should be confirmed with immunohistochemical staining [21]. Although DFSP has been shown to express the CD34 antigen and is one of the means of identifying the tumor, nodular areas of DFSP may have more sparse/variable expression of CD34 [22]. Moreover, fibrosarcomatous DFSP tumors may be negative or only weakly positive for CD34 and this represents an immunohistochemistry pitfall [23].

Interestingly, one patient with a DFSP of the left anterior shoulder with fibrosarcomatous change had a non-contiguous subcutaneous mass on the left posterior shoulder biopsied at the time of her Mohs procedure that resulted in a spindle cell lipoma. A previous case report noted a 48-year-old man with DFSP with fibrosarcomatous change and multiple spindle cell lipomas [24], and given the common denominator of CD34+ interstitial dendritic cells, this indicates that there may be a common cellular progenitor.

As mentioned in the results, a split-thickness skin graft was utilized to close a large defect on the sternum and an advancement flap was used to close a large defect on the glabella/central forehead. A DFSP of the sternal chest with a deep, ovoid, and wide final defect was recently reported that was closed using bilateral island pedicle flaps, with the leading edges sutured together centrally in a spiral fashion [25]. This resulted in a satisfactory cosmetic result and demonstrates that there are multiple techniques available for closing such a surgical wound. Likewise, other methods for closure of large central forehead reconstruction after DFSP removal have been reported. This includes an inferior hatchet rotation flap with primary closure of the donor site [26] and tissue expansion followed by bilateral advancement flaps and subsequent running Z-plasty upon scar revision [27]. Neither of these cases had the extent of glabella involvement as our case as they were slightly more superiorly located on the central forehead.

Limitations of this study include a short mean interval for follow-up, as approximately 25% of DFSP recurrences do not occur until five years or longer after surgical removal of DFSP [28]. Additionally, several patients in our review had less than one year of follow-up time between their surgical removal of DFSP and the end of our study period, which is an inadequate period to assess for long-term disease-free survival.

## Conclusions

Given the rarity of DFSP, the additional 15 cases of DFSP treated with MMS presented in this article provide additional data for the efficacy of MMS for its treatment. In our cohort, we observed that there was no recurrence of DFSP following MMS with a mean follow-up interval of 22.4 months.

Our experience also highlights closure techniques for large, ovoid defects of the glabella/central forehead and sternum. A large, deep ovoid defect of the glabella/central forehead can be closed using a unilateral advancement flap with a Burow’s triangle removed from the ipsilateral eyebrow. A split-thickness skin graft may be used with satisfactory cosmetic results even in very wide and deep ovoid sternal chest defects. Moreover, a split-thickness skin graft allows for easier and earlier recognition of local recurrence.
